# Sources of variability in human communicative skills

**DOI:** 10.3389/fnhum.2012.00310

**Published:** 2012-11-22

**Authors:** Inge Volman, Matthijs L. Noordzij, Ivan Toni

**Affiliations:** ^1^Donders Institute for Brain, Cognition and Behaviour, Radboud University NijmegenNijmegen, Netherlands; ^2^Behavioural Science Institute, Radboud University NijmegenNijmegen, Netherlands; ^3^Department of Cognitive Psychology and Ergonomics, University of TwenteEnschede, Netherlands

**Keywords:** social cognition, joint action, tacit communication game, interactive intelligence, cooperation

## Abstract

When established communication systems cannot be used, people rapidly create novel systems to modify the mental state of another agent according to their intentions. However, there are dramatic inter-individual differences in the implementation of this human competence for communicative innovation. Here we characterize psychological sources of inter-individual variability in the ability to build a shared communication system from scratch. We consider two potential sources of variability in communicative skills. Cognitive traits of two individuals could independently influence their joint ability to establish a communication system. Another possibility is that the overlap between those individual traits influences the communicative performance of a dyad. We assess these possibilities by quantifying the relationship between cognitive traits and behavior of communicating dyads. Cognitive traits were assessed with psychometric scores quantifying cooperative attitudes and fluid intelligence. Competence for implementing successful communicative innovations was assessed by using a non-verbal communicative task. Individual capacities influence communicative success when communicative innovations are generated. Dyadic similarities and individual traits modulate the type of communicative strategy chosen. The ability to establish novel communicative actions was influenced by a combination of the communicator's ability to understand intentions and the addressee's ability to recognize patterns. Communicative pairs with comparable systemizing abilities or behavioral inhibition were more likely to explore the search space of possible communicative strategies by systematically adding new communicative behaviors to those already available. No individual psychometric measure seemed predominantly responsible for communicative success. These findings support the notion that the human ability for fast communicative innovations represents a special type of complex collaborative activity.

## Introduction

Human communication relies heavily on complex skills acquired early in life (i.e., language), but we are also endowed with the ability to build new communicative systems from scratch when necessary. Dramatic examples of the latter ability are “home-sign” systems that can be developed by deaf children of hearing parents who have been deprived of access to conventional language (Goldin-Meadow, 2003; Senghas et al., 2004; Sandler et al., 2005). More mundane and pervasive examples are given by daily-life situations where we can communicate without any pre-existing conventions, as when signaling to others out of earshot or without a common idiom. It has been argued that this ability to infer each other's intentions during interactions is not limited to special and contrived situations, or to the establishment of new communicative systems; rather, this ability represents an interactional intelligence which is one of the hallmarks of human cognition (Levinson, 1995).

Early descriptive studies of dialog (Clark and Carlson, 1982; Clark, 1996) as well as more recent systematic investigations (Galantucci, 2005; Selten and Warglien, 2007; Newman-Norlund et al., 2009; de Ruiter et al., 2010) indicate that human communicators can readily create a new shared semiotic system under a variety of constraints. Yet, it is also evident that there is great variation in the manner and the efficiency with which different pairs solve the same communicative challenge (Clark, 1996; Galantucci, 2005; de Ruiter et al., 2010). The aim of the present study is to characterize psychological sources of inter-individual variability in communicative skill, operationalized as the ability to build a new shared communication system. This study was triggered by the suggestion that the large inter-subject variability in successfully setting up a new communication system might be related to a specific trait, namely the co-operative attitude of individuals (Steels, 2006). However, it is also conceivable that, in the specific context of communicative interactions based on visuospatial material (e.g., Galantucci, 2005; de Ruiter et al., 2010), communicative success could also be explained by the ability to deal with complex spatial problems. In this study, we systematically investigate those possibilities using measures of empathizing and systemizing abilities (Wheelwright et al., 2006), affinity for complex thought (Cacioppo et al., 1984) and capability to deal with complexity (Raven, 2000). We reasoned that inter-individual variability in communicative skills could emerge from either domain-general or domain-specific cognitive abilities, and be driven by either complementary or overlapping cognitive profiles of the communicators. First, if variability in communicative skills is related to general-purpose cognitive abilities, then abilities deployed across a variety of cognitive domains should account for a large portion of inter-individual variability in communicative skill. Alternatively, the ability to build a new shared communicative system might rely on a specialized communicative skill, previously labeled as “interactional intelligence” (Levinson, 2006) or “cultural intelligence” (Herrmann et al., 2007), a competence also studied in recent experimental work on the evolution of shared communicative systems in humans (Kirby et al., 2008; Scott-Phillips et al., 2009, 2012). In this perspective, inter-individual variations in communicative skill would be only marginally related to other general-purpose cognitive abilities, but share some sources of variance with social abilities required for engaging in collaborative activities (Melis et al., 2006). Second, given that communication is a joint construct of interacting agents, it appears relevant to examine how the psychometric profiles of each communicator in a pair influence communicative performance. For instance, there could be dissociable individual traits that significantly support successful communication. Alternatively, it might be that the success in establishing new shared communication systems is not determined by the individual abilities *per se*, but by the overlap between the abilities of individuals within a communicative setting.

In this study, we quantified inter-individual variations in communicative skill by means of a controlled and validated experimental setting, the Tacit Communication Game (TCG) (Newman-Norlund et al., 2009; Noordzij et al., 2009; de Ruiter et al., 2010). The TCG is an online, interactive, non-linguistic communicative task in which two players have to jointly recreate a simple goal configuration of two geometrical objects (e.g., circles and triangles) located in a three by three grid (Figure 2). The crucial element of this game is that only one player (the sender) initially sees this goal configuration, while the other player (the receiver) does not. Therefore, solving the game requires that the sender communicates to the receiver where and how his object should be positioned in the grid. This game allowed us to distinguish the creation of new communicative behaviors from the utilization of pre-established conventions. More precisely, subject pairs started by solving a set of communicative problems (labeled as OLD trials) such that every pair had established a successful, shared communicative rule. Afterward, these OLD trials were intermixed with NEW communicative problems, i.e., communicative problems in which new shared conventions needed to be established. This experimental design allowed us to examine individual differences specifically related to the ability to generate new, non-linguistic communicative conventions, having controlled for the ability to implement and exploit previously established conventions in the same task settings. Namely, individual differences in performance of the TCG can be quantified by the speed and accuracy with which participant pairs jointly succeed in matching the goal configuration during the NEW trials. The behavior of the senders can also be further classified according to the type and number of strategies they use to communicate to the receiver. These strategies can then be classified in terms of their success. For instance, it is possible that two pairs achieve similar communicative success by using very different communication strategies, or by varying their strategies in different manners. Therefore, we examine whether particular communication styles can be associated with specific individual traits. Finally, alignment accounts of dialog predict that communicative skill is mainly determined by the overlap between the situation models of the interactants (Pickering and Garrod, 2004). Therefore, we considered the overlap between individual traits of a communicative pair, comparing TCG performance with the absolute difference between the score of the sender and receiver within a pair (“mismatch score”) across a set of psychometric measures. If novel communicative conventions are more readily established between individuals that are more alike, then there should be a negative relation between this mismatch value and the performance measures.

We considered a set of parameters that have been previously validated and used to characterize various cognitive and social abilities. These parameters were chosen on the basis of the following considerations. First, when solving a communicative problem, people need to identify not only what is ambiguous according to their viewpoint, but also what is ambiguous to their communicative partner. These might be different components of the problem. This aspect of interactive intelligence resembles abilities that have been proposed in the human emotional domain. For instance, empathy refers to the ability to identify other's feelings and emotions and to respond to these in an appropriate way. It could be that highly empathic individuals are better able to establish new communicative conventions. The empathizing quotient (EQ) is one way to measure empathy (Baron-Cohen and Wheelwright, 2004). Another empathy scale is the interpersonal reactivity index (IRI) of Davis (1980). This questionnaire consists of four subscales, each considered to capture an important aspect of empathy. Systemizing abilities have been proposed as being somehow orthogonal to empathy, and these abilities can be measured using the systemizing quotient revised (SQ-R) (Wheelwright et al., 2006). Interpreting the behavior of others through a set of rules (i.e., using a systemizing approach) might be counterproductive when establishing communicative conventions, and this would result in a worse TCG performance. Second, resolving a communicative ambiguity in the TCG often requires the generation of novel semiotic conventions. This implies an understanding that a new situation has actually arisen, requiring to implement communicative actions that fall outside an existing repertoire. The speed and extent of this realization might be related to the subjects' affinity for understanding intentions, as measured using the need for cognition scale (NCS) (Cacioppo et al., 1984; Evans et al., 2003). This suggests that individuals high in need for cognition might appreciate communicative ambiguities earlier and thus be more successful in establishing novel communication systems. A related cognitive trait relevant for communication could be the ability to recognize patterns within a given problem, as captured by Raven's progressive matrices (Raven, 2000). Third, we considered two general psychometric measures of cognitive style. The cognitive style indicator (COSI) is a questionnaire that measures different styles in planning, knowing, and creating (Cools and Van den Broeck, 2007). Behavioral inhibition and behavioral activation scales (BIS/BAS) (Carver and White, 1994) index motivational influences, e.g., sensitivity to punishment (leading to anxiety about conveying the wrong message to the communicative partner) and to reward (enhancing the drive toward generating situations in which positive feedback prevails).

## Materials and methods

### Participants

**Figure 1:**
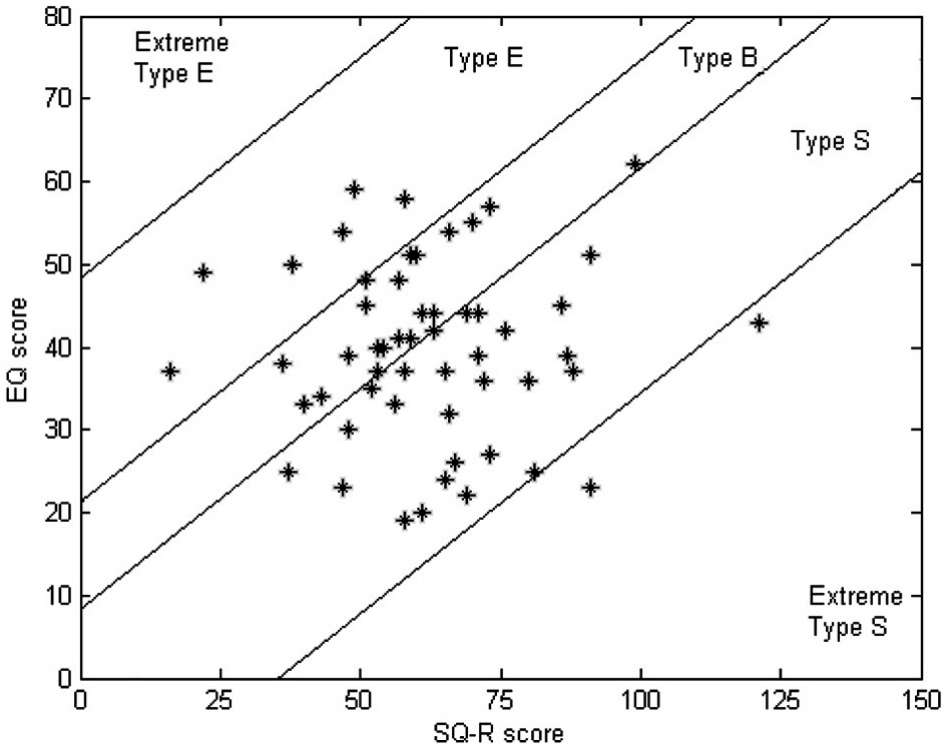
**The EQ and SQ-R scores of the participants with the boundaries of different types according to Wheelwright et al. (2006)**.

We tested 54 participants. They were right-handed male students (18–27 years), with normal or corrected to normal vision. This group of participants was selected from a larger pool of 285 subjects, contacted by means of e-mails and flyers, who completed two questionnaires, the EQ and SQ-R (Wheelwright et al., 2006), on an online website. The information provided by the students at this website was protected according to the Dutch Personal Data Protection act. If a student had *prior* knowledge about the questionnaires, he was excluded from the selection. Subjects were selected according to their EQ and SQ-R scores, in order to obtain a group of participants uniformly spread over the EQ and SQ-R spectrum. The EQ and SQ-R scores of the participants are shown in Figure 1.

**Figure 2:**
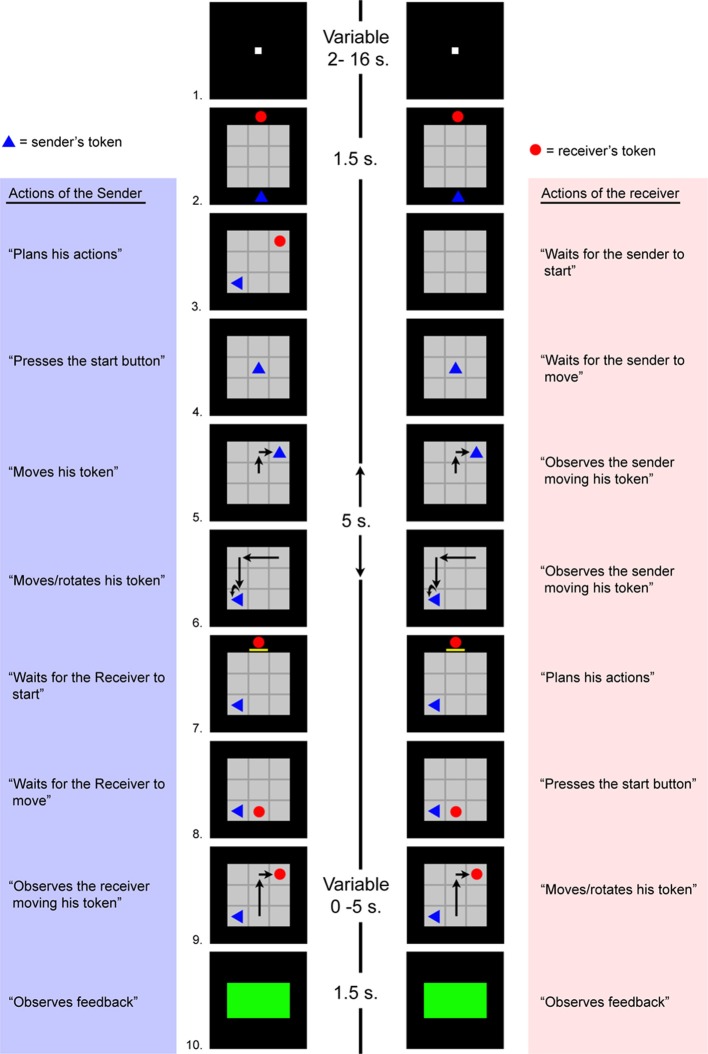
**A timeline corresponding to the sender's and receiver's observations and actions during the OLD trials.** The sender and the receiver saw the images presented in the left and right column, respectively. A trial started with a fixation point presented on the screen (#1). After 2 s the game board and the tokens appeared (#2). Then the goal configuration was shown to the sender, and not to the receiver. The goal configuration consisted of two tokens inside the game board (#3). The sender had unlimited time to look at the goal configuration and plan his moves. After the sender pressed the start button, all tokens disappeared and the sender's token appeared in the center of the game board (#4). The sender had 5 s to move his token within the game board (#5, 6). A yellow bar under the receiver's token indicated that the 5 s had passed and the receiver could start to move (#7). The receiver had unlimited time to plan his moves. After the receiver pressed his start button, his token appeared at a random location on the game board (with the exclusion of the goal positions of either sender or receiver) (#8). After the first move, the receiver had 5 s to move within the game board (#9). When the receiver finished within 5 s, he could end his turn by pressing the start button. The participants received visual feedback about their performance (#10). A green rectangle indicated a correct match with the goal configuration, a red rectangle an incorrect match.

The participants were assigned into 27 communicative pairs, arranged in order to cover different combinations of EQ and SQ-R scores. Because type S contained the largest group and we wanted to obtain a good spread, we further divided type S into two sub-groups by introducing an extra boundary in the middle of type S. Pairs were then generated by randomly choosing participants from two different types out of the five categories. All participants gave informed consent according to the institutional guidelines of the local ethics committee (CMO region Arnhem-Nijmegen, The Netherlands). The participants received a financial payment or course credits for their participation.

### Questionnaires

We considered seven psychometric questionnaires, requiring forced-choice responses. Two questionnaires (EQ and SQ-R) were administered through a website, during subjects selection (see above), one to six months before performance of the TCG (Part I). Two questionnaires (Raven, NCS) were administered in the laboratory immediately after performance of the TCG (Part II). Three questionnaires (IRI, COSI, BIS/BAS) were administered at home, approximately 8 months after performance of the TCG (Part III). Part III of the experiment was conducted by forty participants (20 senders, 15 complete pairs) who returned the questionnaires.

Details on the construction of the EQ and SQ-R can be found in Baron-Cohen and Wheelwright (2004) and Wheelwright et al. (2006). The NCS consisted of 18 statements. Details on the construction of the NCS can be found in Cacioppo et al. (1984). All three questionnaires were translated to Dutch. The Raven's test (Raven et al., 1995) consisted of 36 items and the participants had 20 min to work on them. With the use of an example item, it was explained to the participants that they needed to find the missing design of a particular sequence of designs. Details on the construction of the IRI can be found in Davis (1980), of the COSI in Cools and Van den Broeck (2007) and of the BIS/BAS in Carver and White (1994).

### Procedures and materials

The experiment was structured in three-parts. Part I was the web-based subject selection (see above). Part II took place at the Donders Institute for Brain, Cognition and Behaviour (Nijmegen, The Netherlands) and it consisted of a TCG training session, a TCG testing session, and a psychometric session, in this order. Part III involved completing three more questionnaires (IRI, COSI, BIS/BAS; see above). In the following sections we focus on the procedures of Part II. During the TCG training session (duration: 30 min), subjects were familiarized with the TCG. During this session, each communicative pair generated and learned a communicative rule for solving a set of TCG problems (see below). During the TCG testing session (duration: 40 min), each communicative pair solved both learned and new TCG problems. During the psychometric session (duration: 30 min), subjects were administered the Raven's test and the NCS in consecutive order. Below we elaborate on the procedures followed during the TCG training and testing sessions. In both sessions, the participants could not see or hear each other. Each participant used Logitech hand-held controllers to move an object shown on a computer monitor. The four face buttons of the controller were used for movements to the left, right, up, and down, two shoulder buttons were used to rotate the token clockwise and counter-clockwise, and another shoulder button was used as a start and end button. The TCG was programmed using Presentation version 10.1 and was run on a Windows XP personal computer.

#### TCG training session

The TCG training session was structured in three sub-sessions, sequentially presented. First, the participants were individually familiarized with the experimental setup (40 trials). Namely, each participant saw a blue triangle (the target) with a random rotation at a certain location on the game board. After the participant pressed the start button, the target disappeared from the game board, and a triangle that pointed upward appeared in the center of the game board (player's token). The participant had to position his token in the location and orientation of the target previously shown, by pushing the appropriate buttons on the hand-held controller. After the participant matched the target with his token, a new target was shown, in a pseudo-randomly chosen position and orientation on the game board.

Second, the participants were jointly introduced to the basic procedures of the TCG (10 trials). Each participant of a communicative pair was assigned the role of either sender or receiver, and he kept this role during the remainder of training and testing sessions. During this training sub-session, the participants were asked (by means of written instructions) to use their tokens to match the targets configuration shown on the game board (see Figure 4 for more details). On each trial, there were two targets, one for each participant's token. Each participant could control only one token and the color of that token remained the same throughout the experiment, blue for the sender, red for the receiver. The tokens could have a circular, triangular, or rectangular shape. Crucially, during this training sub-session, both participants could see the targets configuration.

Third, the participants were jointly introduced to the communicative aspects of the TCG (at least 25 trials). This training sub-session was identical to the second sub-session, apart from one important difference, namely only the sender could see the targets configuration. Each communicative pair was informed about this change with written instructions. This change meant that, to successfully complete a trial, the sender had to communicate to the receiver the location and in some cases the orientation of the receiver's token. Given the structure of the TCG, the sender could communicate this information to the receiver only by moving his own token around the game board. The sender was encouraged to think how to do so before pressing the start button.

If a communicative pair made a mistake during the last ten trials of this training sub-session, they had to complete ten extra trials until they had performed ten correct trials sequentially. This type of communicative problems was labeled as OLD, since by the end of this training sub-session each communicative pair was successful in solving these problems with a consistent communicative strategy.

#### TCG testing session

To investigate the establishment of new shared communicative actions, we compared a situation in which communicative rules were already established (OLD problems) with a situation in which a communicative rule was yet to be established (NEW problems). During the testing session the pair played a version of the TCG consisting of such OLD and NEW trials. The old trials of this session were similar to the OLD trials of the third part of the training session. The similarity was based on the fact that the same communicative strategy could be applied. In contrast, the NEW trials entailed different problems. Namely, the sender had to indicate both location and rotation of the receiver's token with his own token, although the shape of the sender's token contained less rotation possibilities than the shape of the receiver's token (see Figure 6). This forced the pair to invent novel communicative strategies in order to have a successful trial. There were four different shape combinations for the OLD and for the NEW problem. These shape combinations made it possible to create different situations that had to be communicated. The differences between the combinations were created by giving the players different tokens and by letting the triangle, when using this token, point to the inside or outside of the game board. For instance, when the receiver's triangle is pointing to the inside of the game board, the sender could move his token to the neighboring grid following the pointing direction of the receivers token. If the receiver's triangle was pointing to the outside of the game board, the sender could not use the strategy described above to indicate the rotation of the token. In this situation another strategy is needed to unambiguously signal the goal configuration to the receiver.

At the start of the testing session, the players received a short written instruction with a summary of the most important game features experienced during the previous training session. These points were: only the sender can see the goal configuration; after pressing the start button you have 5 s to move; both location and rotation of the token need to be correct; try to be quick, but more importantly try to get as many trials correct as possible; press the end button after you have finished moving your token (for the receiver only).

**Figure 3:**
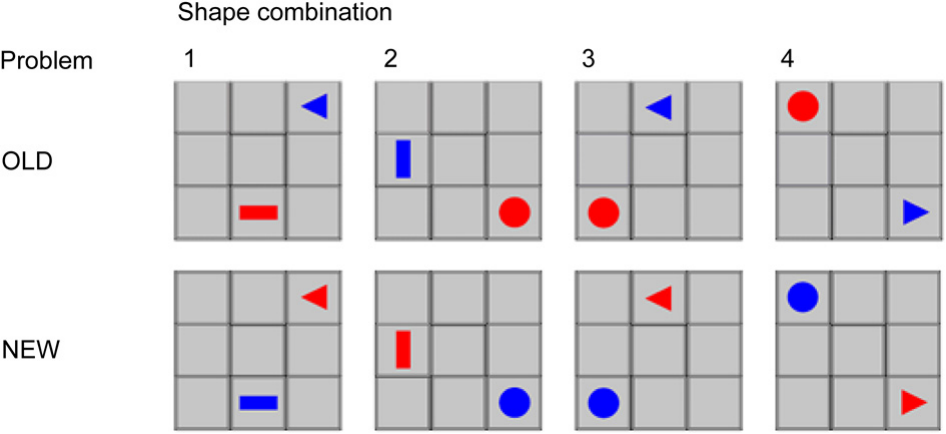
**Examples of goal configurations from different communicative problems and the corresponding shape combinations.** The tokens are matched in shape for OLD and NEW problems, but with different communicative roles. Note that in shape combination three, the triangle is pointing toward the game board, whereas in shape combination four the triangle is pointing away from the game board.

The experimental session contained 84 trials; half were OLD trials, half were NEW trials. No more than either three OLD or three NEW trials were presented sequentially. For the OLD trials, the presentation of the shape combinations was intermixed. For the NEW trials, there were four shape combinations (Figure 3), presented in succession. When a pair solved four NEW trials from one shape combination consecutively, it was assumed that the pair had developed a consistently successful communicative strategy for that trial type. Accordingly, problems with this shape combination were not presented further. If a pair solved three of the NEW shape combinations, then trials with the fourth shape combination were presented until the end of the testing session.

### Data analyses

#### Psychometric measures

Group differences between psychometric scores of senders and receivers were assessed with a One-Way ANOVA. The relations between the psychometric scores of each subject were investigated by means of bivariate correlation analyses.

The relation between psychometric scores of participants within a pair was quantified by means of a difference score (defined as the absolute value of the difference between sender and receiver scores), an indicator of the similarity of the two individuals that constitute a pair. Lower difference scores reflect larger similarities on that particular psychometric test.

#### TCG performance

For each pair, we considered two indices of TCG performance, i.e., mean accuracy across the testing session, and its rate of change. The mean accuracy of each pair was analyzed using repeated-measures ANOVA (threshold, *p* < 0.05) with problem type as a factor (two levels: OLD and NEW). Change in performance (learning rate) was analyzed using linear regression analyses, with the log transformed trial number as independent variable (i.e., considering change in performance as following a logarithmic profile). For each pair of participants, we calculated the slope of change in performance over trials by considering the beta value of the linear regression between the moving average of accuracy (NEW problems only) and the log transformed trial number. A moving average over four trials was used, but at the end points, where there are less than four datapoints available, a moving average over two trials was used.

We tested for the influence of communicative strategy by using an ANOVA considering the effect of those strategies (categorized as COARSE and REFINED, see section “Results” for a full description) on success rate and frequency of occurrence. Each trial was replayed offline and categorized accordingly. We used repeated-measures ANOVAs to test whether COARSE or REFINED strategies influenced success rate and strategy occurrence.

#### Psychometric relations to TCG performance

To test whether the psychometric scores of the senders and the receivers influence performance and strategy choice during the NEW problems, linear regression analysis were performed. First, only the psychometric scores assessed for all participants (from part I and II) were included. Second, the psychometric scores of part III were included as well (15 complete pairs). The two measures of performance used as dependent variable are the mean accuracy scores and the learning rate of each pair. Strategy choice was defined by the occurrence of each strategy group. An estimate of change in performance (standardized beta value) was obtained by means of linear regression analyses with accuracy (moving average) as dependent variable and trial number (log transformed) as independent variable. The independent variables (psychometric scores) were entered into the linear regression model following a stepwise fashion, meaning that only those independent variables that explained a significant (and unique) part of the variance of the dependent variable were entered into the model. The significant models (*p* < 0.05) are reported.

Overlap/differences in psychometric profiles of participants within each pair were quantified by creating “mismatch values” for each psychometric measure, defined as the absolute difference between the score of the sender and the receiver. Mismatch values of different psychometric scores were then entered in a linear regression model following a stepwise inclusion procedure.

## Results

### TCG performance

One pair was excluded from analyses because of their poor performance on both OLD and NEW trials (79%, 0% correct, respectively), indicating an inability in establishing and maintaining a communicative system, especially on the NEW trials. The idiosyncratic behavior of this pair is described in Box 1.

Box 1 **Case study: What if the receiver does not pick up on novel communicative actions?**

The receiver of one pair did not understand that he had to turn, but at the end of the experiment he did indicate that he knew he could turn. He did not turn his shape in any of the trials. Starting from the first NEW trial, the sender was using different strategies to indicate to the receiver that he needed to turn. The sender used 6 different strategies, but strategy IV was used the most (25 of the 42 times). If the sender had a rectangle he would rotate it, but he also moved along the whole row or column of the game board to indicate a pointing direction.

The receiver had the lowest Raven score of all participants. This corresponds with findings from other pairs, namely pairs composed of a receiver with low fluid intelligence are less effective at establishing a novel communicative system.

**Figure 4:**
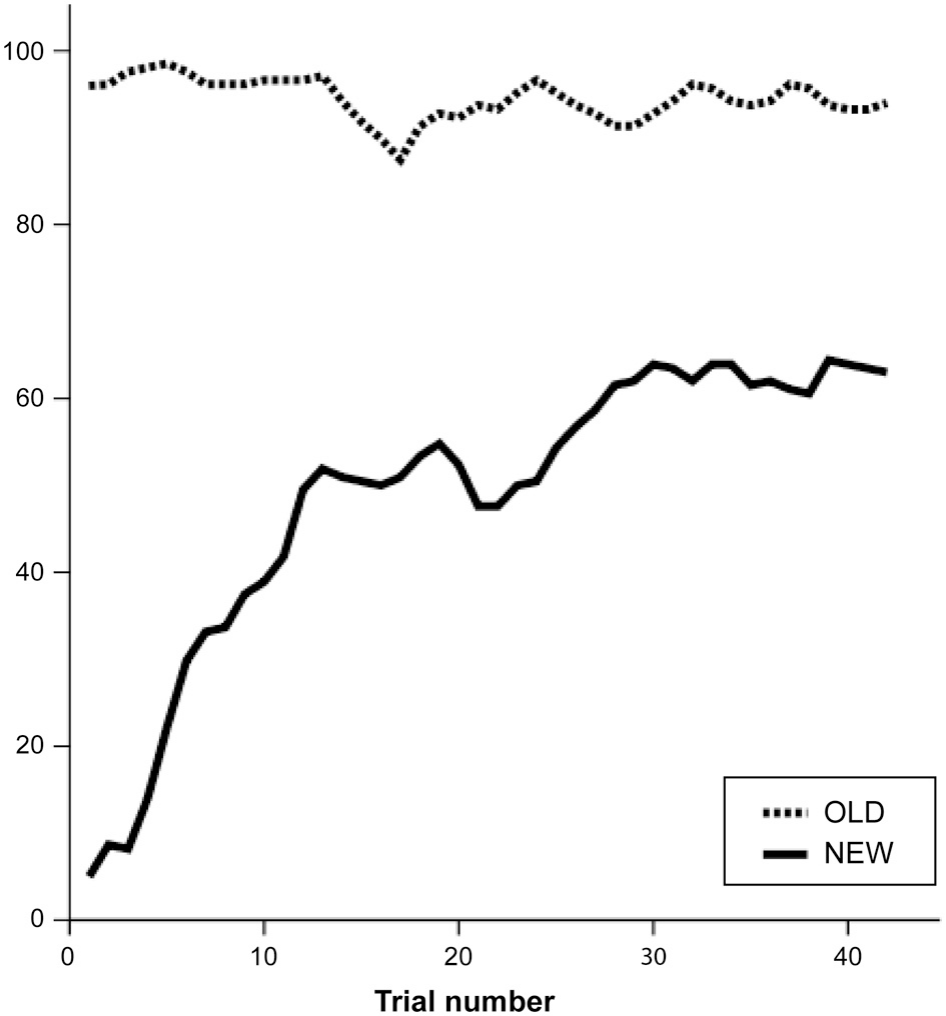
**Timecourse of task performance (accuracy, in %) over all pairs**.

Mean accuracy scores showed a significant effect of problem, *F*_(1, 25)_ = 184.4, *p* < 0.001, with more errors for the NEW (mean = 49% correct, *SE* = 3.5) than the OLD problems (mean = 95% correct, *SE* = 0.9). Figure 4 visualizes the changes in performance during the game. Performance improved when solving NEW problems, *F*_(1, 40)_ = 35.2, *p* < 0.001, according to a logarithmic profile. There was no significant change in performance for the OLD problems, *F*_(1, 40)_ = 1.0, *p* = 0.329.

During the NEW trials the pairs had to develop particular strategies to convey a message about location and rotation of the receiver's shape. These communicative strategies were divided into two main groups: COARSE, in which the desired rotation of the receiver's shape was indicated with little or no information, and REFINED, in which more elaborate movements indicated the rotation. The COARSE group consisted of three strategies: (1) the sender indicated the position of the receiver's shape only, ignoring its rotation; (2) the sender used the direction in which he moved away from the middle of the game board (sender's start position) to the receiver's target position as a marker for the desired orientation of the receiver's shape; (3) the sender used the direction in which he moved away from the receiver's target position to his own target position as a marker for the desired orientation of the receiver's shape. The REFINED group consisted of five strategies that explicitly indicate the movement and rotation of the token; (4) the sender moved to the receivers location, after which he moved one square in the pointing direction and back to indicate the desired rotation; (5) the sender first indicated the desired rotation of the receiver's token by moving in that direction (and back to the starting position) before moving to the receiver's location; (6) when the sender had a rectangle token, he indicated the desired rotation by rotating his rectangle the desired amount of rotations; (7) after moving to the receiver's desired position, the sender indicated rotation by moving his token along the whole row or column of the receiver's goal position; (8) the sender indicated rotation by imitating a rotation, namely moving his token along a square across the whole board (e.g., one square up, one to the right, one down and one to the left. We also considered two additional, independent categories; (9) other idiosyncratic strategies observed for a few trials only; (10) no definite strategy.

**Figure 5:**
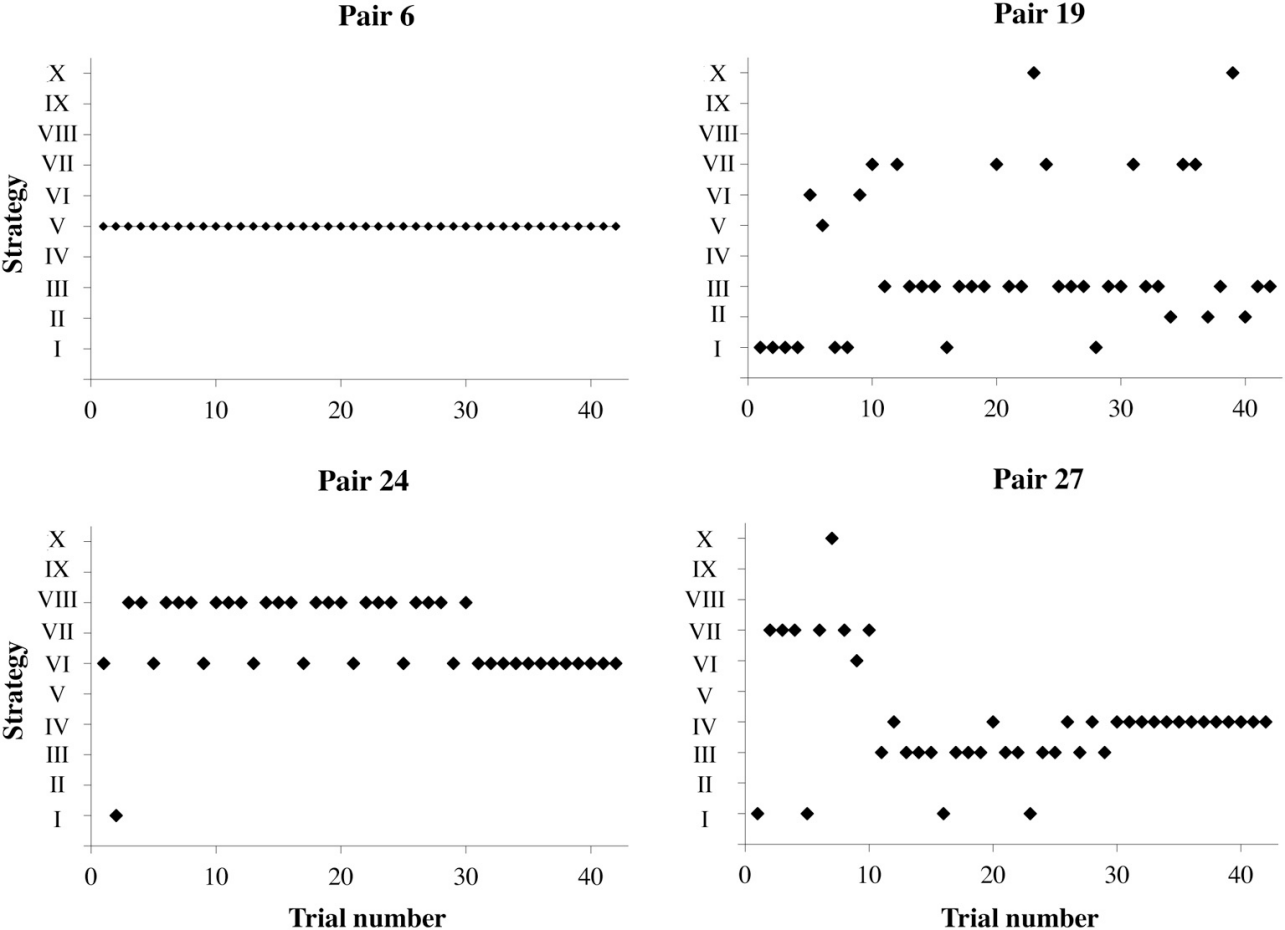
**The different strategies used by four pairs with respect to the NEW problem.** The Roman numerals indicate different strategies, as described in the main text.

During the game, different pairs used different strategies, in different proportions, as illustrated in Figure 5 for a few representative pairs. For instance, Pair 6 used a single strategy, consistently and successfully. Pair 19 had difficulty in converging on a single strategy. Other two pairs showed intermediate variability.

Some strategies had a higher success rate than others, and there were also differences in the number of times a strategy was used (Table 1). There was a significant correlation between accuracy and occurrence for either strategy group (COARSE: *r* = 0.577, *p* = 0.003; REFINED: *r* = 0.567, *p* = 0.008). This shows that the higher the use of a given strategy, the better the pair's performance was. If the pairs only used the COARSE or only the REFINED convention, instead of both, then they solved more NEW trials, *r* = 0.658, *p* < 0.001.

**Table 1:** **Mean and standard error of accuracy and frequency of occurrence for different strategies**.

**Strategies**	**Mean ACC**	**Occurrence %**
**COARSE**		
I	0.20	17.0
II	0.93	3.7
III	0.91	3.2
**REFINED**		
IV	0.53	41.0
V	0.93	3.9
VI	0.39	10.0
VII	0.39	6.4
VIII	0.76	1.9
**OTHER**		
IX	0.67	8.0
X	0.09	4.9

Strategies are described in the main text.

### Psychometric relations to TCG performance

**Figure 6:**
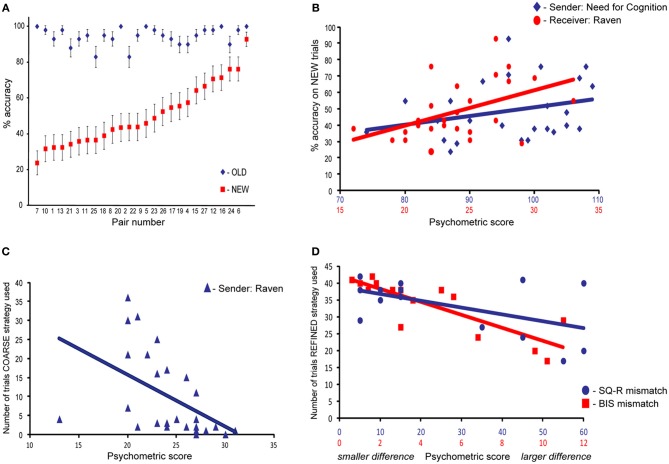
**(A)** Mean accuracy scores for OLD and NEW problems for each pair of participants. **(B)** Scatterplot of the relation between performance during NEW trials, senders' Need for Cognition score, and receivers' Raven score. **(C)** Scatterplot of the relation between frequency of using a COARSE strategy and senders' Raven scores. **(D)** Scatterplot of the relation between frequency of using a REFINED strategy, within-pairs SQ-R mismatch score, and within-pairs BIS mismatch score.

Figure 6A shows that performance on the OLD problems was consistently stable across pairs, whereas performance on the NEW problem changed from pair to pair. This paper assesses whether this considerable inter-subject variability can be accounted by different cognitive traits. The sender's NCS scores and the receiver's Raven scores accounted for a significant portion of variance in TCG performance [*R*^2^_(23)_ = 0.286, β = 0.367, *p* = 0.042; β = 0.516, *p* = 0.006, respectively; see Figure 6B]. This indicates that for the NEW problems, the best performing pairs were composed of a sender with high's need for cognition and a receiver with high fluid intelligence. A comparison between change in performance and psychometric scores showed that the higher the receiver's score on the Raven's test, the faster performance increased on NEW problems, *R*^2^_(24)_ = 0.155, β = 0.434, *p* = 0.027. This indicates that the higher the receiver's fluid intelligence, the quicker the pair established a novel communication. Although, Figure 6C might suggest the presence of an outlier, descriptive analyses do not support this intuition, and excluding that datapoint (Raven score of 13) from the analysis strengthen the statistical inference (*R*^2^_(23)_ = 0.436, β = −0.678, *p* < 0.001). Furthermore, even though the *R*^2^ of these analyses might appear numerically small, in fact a correlation coefficient (*R*) around 0.10 is considered to reflect a small association, and 0.30 a moderate correlation (Cohen, 1988).

There was no significant relation for the sender. The pair's (dis)similarity did not influence overall performance or rate of change.

To investigate the influence of individual cognitive traits on usage of different communicative strategies, we considered the occurrence of COARSE and REFINED strategies. There was a negative relation between the Raven score of the senders and their use of COARSE strategies, *R*^2^_(24)_ = 0.205, β = −0.486, *p* = 0.012; see Figure 6C. In other words, senders with high fluid intelligence rarely used COARSE strategies.

Finally, we investigated the influence of the overlap in psychometric profiles of participants within each pair on the usage of different communicative strategies. This analysis was based on the psychometric measurements of part III, completed by 15 pairs only. Participants mismatch on the SQ-R and BIS scores decreased the chances of using a REFINED strategy (SQ-R: *R*^2^_(12)_ = 0.778, β = −0.743, *p* < 0.001; BIS: *R*^2^_(12)_ = 0.787, β = −0.377, *p* = 0.011). In other words, smaller within-pairs differences in systemizing abilities and in sensitivity to negative cues lead to increased frequency of REFINED strategies.

## Discussion

The aim of this study was to investigate the psychological traits leading to inter-subject variation in communicative skills. We operationalized communicative skill as the ability to build shared communicative innovations. We describe qualitative and quantitative indexes of communicative performance in pairs of participants engaged either in applying previously established communicative conventions, or in establishing new shared conventions. Three observations indicate that the experimental procedures were effective in capturing communicatively relevant variability in subjects' performance. First, when faced with new communicative problems, subjects' pairs progressed from communicative failure (early in the experiment) toward mutual understanding (late in the experiment). This improvement in communicative performance occurred despite the expansion of the set of problems faced by the participants, as NEW trials were progressively introduced, and previously established communicative conventions might have become ineffective. Second, there were large differences in the ability of the different pairs to establish shared communicative strategies. Some pairs quickly established a novel successful communicative strategy, while others had more difficulty in doing this. Third, pairs differed in their inclination to change communicative strategies during the course of the experiment, a sign of mutual adjustment during social interactions (Clark, 1996). Accordingly, we could test whether these differences in communicative skill were related to cognitive traits, quantified through measures of empathizing and systemizing abilities, behavioral inhibition, fluid intelligence, need for cognition, and cognitive style.

There are three main findings in this study. First, the ability of a pair to successfully establish novel communicative actions was influenced by a combination of the sender's need for cognition (NCS) and of the receiver's Raven's score. It is known that the learning strategies of individuals with high NCS are more flexible by virtue of being less biased by surface information (Cacioppo et al., 1996; Ruiter et al., 2004). Here we show that this cognitive trait is beneficial for supporting the introduction of a new communicative system, possibly in relation to finding a deep structure robust to the continuously changing problems of the NEW trials. In contrast, individuals with low NCS scores have reduced intrinsic motivation to solve cognitive challenges and are more likely to rely on others to find meaning in events and stimuli (Cacioppo et al., 1984; Evans et al., 2003). These individuals have more difficulties in introducing new communicative strategies, as required from senders in the current experimental setting. A slightly different set of cognitive traits were important to account for communicative performance in receivers, and in particular on the efficiency with which a new communication system was established. Participants in this role were particularly effective when they had a good fluid intelligence, as indexed by the Raven questionnaire (Carpenter et al., 1990). Senders with high Raven scores were also more likely to generate refined communicative strategies. It appears that individuals with high Raven scores are better equipped to generate and find analogical mappings between actions and their underlying communicative intentions.

Second, pairs with comparable systemizing abilities or behavioral inhibition were more likely to use refined communicative strategies. More precisely, pairs with high systemizing scores and particularly averse to negative feedback appear more likely to explore the search space of possible communicative strategies by systematically adding new communicative behaviors to the available conventions, i.e., safely building on pre-existing behaviors rather than violate pre-existing conceptual pacts (Brennan and Clark, 1996) by introducing subtle modulations of those behaviors.

Third, measures of empathy and reward-related tendencies (BAS) were not able to account for significant portions of inter-subject variability in communicative performance. This negative result complement the finding of a previous study that, using a similar communicative challenge, reported a relation between empathy scores and audience design abilities (Newman-Norlund et al., 2009). Taken together, these results suggest that while pro-social attitudes (approximately indexed by empathy) might provide the motivational drive necessary for adjusting a communicative behavior to a given agent (Tomasello, 2008), other general-purpose cognitive abilities (approximately indexed by systemizing scores) might provide the computational tools necessary to cope with the complexity of human communication (Van Rooij et al., 2011).

### Interpretational issues

It might be argued that the findings of this study are not relevant for understanding how humans try to modify the mental state of another agent according to their intentions. For instance, the same findings might have been obtained when the communicator were interacting with an artificial agent producing a pre-defined set of behaviors. In fact, collateral evidence clearly indicate that subjects engaged in this game consider the mental state of the other participant, as indicated by the presence of audience design effects (Newman-Norlund et al., 2009), elaborated repair mechanisms following communicative failures (Blokpoel et al., 2012), sensitivity to the knowledge of the other participant (de Ruiter et al., 2010), and involvement of brain areas associated with mentalizing during planning and understanding the communicative actions used in this game (Noordzij et al., 2009; Stolk et al., submitted). It might also be argued that this experimental setup lacks a naturalistic interactive component, e.g., the continuous multimodal reciprocal feedback experienced during face-to-face social interactions. In fact, the relatively slow dynamics of the task is explicitly designed to capture one crucial element of communicative interaction, namely sharing meanings by producing and interpreting behaviors extended over several seconds. However, it remains to be seen whether the present results, obtained in the context of this highly controlled experimental setup, generalize to other communicative materials (e.g., linguistic and/or gestural), and to situations where communicative roles can be frequently exchanged, as during natural dialog.

## Conclusion

We show that inter-individual variability in communicative skills is partially accounted for by a number of cognitive traits. Individual capacities influence communicative success, when communicative innovations are generated, while dyadic similarities as well as individual traits modulate the type of communicative strategy chosen. Given that no individual psychometric measure was predominantly responsible for communicative success, we infer that general-purpose cognitive abilities are unlikely to fully account for human communicative skills. Existing indexes of cognitive abilities fail to adequately capture elements of those skills. Accordingly, it appears relevant to develop novel and quantitative indexes of communicative skills, analogous to those recently introduced to quantify social skills in children and non-human primates (Herrmann et al., 2007), in order to measure how the ongoing interaction between two adaptive agents can generate relevant joint constraints (see also Riley et al., 2011). The TCG used in this study might provide a simple platform for quantifying communicative skills in humans. For instance, it could be used to assess communicative capabilities of patients with limited access to syntactic and/or semantic knowledge (e.g., Autism Spectrum Disorders, Williams Syndrome, Aphasia; see Willems et al., 2011). The task might also be adapted to investigate the development of communicative capabilities in human infancy (Stolk et al., submitted), and to measure neurophysiological signals under experimentally controlled yet communicatively relevant conditions (Newman-Norlund et al., 2009; Noordzij et al., 2009, 2010).

### Conflict of interest statement

The authors declare that the research was conducted in the absence of any commercial or financial relationships that could be construed as a potential conflict of interest.
